# Mechanistic studies of Gemcitabine-loaded nanoplatforms in resistant pancreatic cancer cells

**DOI:** 10.1186/1471-2407-12-419

**Published:** 2012-09-22

**Authors:** Anne-Laure Papa, Sudipta Basu, Poulomi Sengupta, Deboshri Banerjee, Shiladitya Sengupta, Rania Harfouche

**Affiliations:** 1 BWH-HST Center for Biomedical Engineering, Harvard Medical School, 65 Landsdowne Street, Cambridge, MA, 02139, USA; 2 Channing Laboratory, Department of Medicine, Brigham and Women's Hospital, Harvard Medical School, 65 Landsdowne Street, Cambridge, MA, 02139, USA; 3 MIT Division of Health Sciences and Technology, Harvard Medical School, 65 Landsdowne Street, Cambridge, MA, 02139, USA; 4 Dana Farber Cancer Institute 450, Brookline Avenue, MA, 02215, USA

**Keywords:** Gemcitabine, Pancreatic cancer, Liposome, Poly(lactic-co-glycolic acid), Transmission electron microscopy

## Abstract

**Background:**

Pancreatic cancer remains the deadliest of all cancers, with a mortality rate of 91%. Gemcitabine is considered the gold chemotherapeutic standard, but only marginally improves life-span due to its chemical instability and low cell penetrance. A new paradigm to improve Gemcitabine’s therapeutic index is to administer it in nanoparticles, which favour its delivery to cells when under 500 nm in diameter. Although promising, this approach still suffers from major limitations, as the choice of nanovector used as well as its effects on Gemcitabine intracellular trafficking inside pancreatic cancer cells remain unknown. A proper elucidation of these mechanisms would allow for the elaboration of better strategies to engineer more potent Gemcitabine nanotherapeutics against pancreatic cancer.

**Methods:**

Gemcitabine was encapsulated in two types of commonly used nanovectors, namely poly(lactic-co-glycolic acid) (PLGA) and cholesterol-based liposomes, and their physico-chemical parameters assessed *in vitro*. Their mechanisms of action in human pancreatic cells were compared with those of the free drug, and with each others, using cytotoxity, apoptosis and ultrastructural analyses.

**Results:**

Physico-chemical analyses of both drugs showed high loading efficiencies and sizes of less than 200 nm, as assessed by dynamic light scattering (DLS) and transmission electron microscopy (TEM), with a drug release profile of at least one week. These profiles translated to significant cytotoxicity and apoptosis, as well as distinct intracellular trafficking mechanisms, which were most pronounced in the case of PLGem showing significant mitochondrial, cytosolic and endoplasmic reticulum stresses.

**Conclusions:**

Our study demonstrates how the choice of nanovector affects the mechanisms of drug action and is a crucial determinant of Gemcitabine intracellular trafficking and potency in pancreatic cancer settings.

## Background

Pancreatic ductal adenocarcinoma (PDA) kills 37,680 Americans per year, with a mortality rate of 91%, making it the deadliest of all cancers [1-4]. Since 1998, the nucleoside analogue Gemcitabine has been the drug of choice for treating PDA, often in conjunction with radiotherapy and/or a cocktail of other chemotherapeutics [2,5-7]. However, Gemcitabine only marginally improves lifespan, mainly due to its chemical instability and poor cellular uptake, resulting in an extremely short half-life and bioavailability [8-12]. This translates into frequent administrations of Gemcitabine at high doses, culminating in significant systemic toxicity and associated resistance, thus overshadowing the drug’s promising pharmacological effects. Recent attempts to remedy this problem by trying new drug combinations have not made it past Phase II clinical trials and are very poorly tolerated by the patient, as the lacunae due to the drug’s low half-life, low cellular uptake and high systemic toxicity remain [5,10,13,14]. These conundrums explain why treatment options have remained stagnant for over the past five decades and create an unparalleled need to find a different modality to deliver Gemcitabine to eradicate pancreatic cancer.

Nanotechnology has made exceptional headway in this regard during the past decade, emerging as a revolutionary platform to treat a wide variety of tumors, mainly due to prolonged drug release, as well as increased cell internalization when under 500 nm in diameter [15-21]. As such, we and others have shown that using biodegradable poly(lactic-co-glycolic acid) (PLGA) or liposomal nanovectors to deliver chemotherapeutics results in significant improvement of tumor burden in a wide variety of cancers, including those of the breast and the skin [17,22-24]. Recently, Gemcitabine liposomal nanoformulations have shown promising results in the Laboratory, including prolonged drug release and attenuation of tumor burden [25-27]. However, the rational for choosing the liposomal nanovector for Gemcitabine, as opposed to other nanoformulations, remains unclear. Furthermore, although it has been clearly documented that nanoplatforms increase endocytosis of Gemcitabine, their underlying mechanisms of action once the drug has entered the cell remains uncharacterized [26,28]. Also unknown is whether the type of nanovector used affects this intracellular trafficking of Gemcitabine. Most nanoparticle studies thus far have only alluded to the intracellular delivery of their payload by indirect means, such as by cytotoxicity and apoptotic studies. A more robust analysis can be performed by combining these studies with ultrastructural characterization by transmission electron microscopy [21]. Elucidation of these factors would allow for more robust nanoparticle engineering.

The aims of this study were two-fold. Firstly, to compare whether nanovector type affects the physico-chemical and biological effects of Gemcitabine and secondly, to directly investigate, at the ultrastuctural level, the underlying mechanisms of action imparted by these nanovectors on Gemcitabine trafficking in pancreatic cancer cells, as opposed to free drug. Towards these aims, Gemcitabine was encapsulated in PLGA or liposomal nanovectors, and the resulting nanoplatforms termed *PLGem* and *GemPo*, respectively. Both nanoparticles were around 150 nm in diameter and provided sustained Gemcitabine release for at least a week. Using a human resistant pancreatic cell line [9], we demonstrated that PLGem promoted more cytotoxicity and apoptosis than both GemPo and free Gemcitabine, which translated into strikingly different mechanisms of action at the ultrastructural level.

## Methods

### Materials

All the solvents were purchased from Sigma-Aldrich (St-Louis, MO) and Fisher Scientific (Pittsburgh, PA), unless otherwise noted, and used without further purification. L-α-Phosphatidylcholine (PC), cholesterol (chol) and polyvinyl alcohol (PVA) were obtained from Sigma-Aldrich, whereas 1,2-distearoyl-*sn*-glycero-3-phosphoethanolamine-N-[amino(polyethylene glycol)-2000] (DSPE-PEG-2000) was purchased from Avanti Polar Lipids (Alabaster, AL). The poly(lactic-co-glycolic acid) (M. W. ≈ 4.2 kDa) having a lactic/glycolic molar ratio of 50:50 was purchased from Lakeshore Biomaterials (Birmingham, AL). Gemcitabine hydrochloride was purchased from Tocris (Ellisville, MO).

### Synthesis of Gemcitabine-encapsulated liposomes (GemPo)

Liposome formulations were made up of PC:Chol:DSPE-PEG-2000 (10:5:1 mass ratio). A 4:1 liposome to Gemcitabine mass ratio (e.g. 16:4 mg) was generated using the lipid film hydration technique. Liposome colloidal suspensions (empty liposome and GemPo suspensions) were obtained by dissolving each reagent in dichloromethane, whereas Gemcitabine was dissolved in methanol using rapid stirring. Solvents were removed by rotary evaporator, using a temperature above that of the gel-liquid crystal transition temperature, thus yielding the formation of a lipid film. To ensure the complete removal of any trace of residual solvents, evaporation was continued under complete vacuum for several additional minutes. Lipid films were then hydrated in phosphate buffered saline (PBS), vortexed and subjected to 25 extrusion cycles, above the Tc and using 0.2 μm polycarbonate filters, in order to obtain unilamellar vesicles under 200 nm. Sizing was performed both by dynamic light scattering (DLS) using a Malvern Zetasizer Nano ZS instrument, as well as by transmission electron microscopy (TEM). The Gemcitabine loading in GemPo was determined by UV-visible using a Shimadzu UV-2450 UV-visible spectrophotometer at the wavelength λ = 268 nm. This also allowed us to calculate the encapsulation efficiency (EE) for GemPo as follows:

(1) $$\text{EE}=\left(\text{mass encapsulated Gemcitabine}\right)/\left(\text{mass starting material Gemcitabine}\right)\text{X}\;100$$

### Synthesis of Gemcitabine-encapsulated PLGA nanoparticles (PLGem)

PLGem were generated by combining a PLGA polymer of ≈ 4.2 kDa in a 4:1 mass ratio with Gemcitabine (e.g. 25.00:6.25 mg) using an emulsion-solvent evaporation technique. 25 mg of PLGA was completely dissolved in 1.0 mL of acetone and mixed with Gemcitabine (dissolved in methanol). The entire solution was emulsified into 25 mL of 2% aqueous solution of PVA (80% hydrolyzed, M. W. ≈ 9,000-10,000 Da) by slow injection with constant homogenization using a tissue homogenizer. This mini-emulsion was added to a 100 mL 0.2% aqueous solution of PVA with rapid stirring overnight at room temperature to evaporate any residual solvents. Nanoparticle size fraction was recovered by ultracentrifugation at 90,000 g and the resulting PLGem were lyophilized for 2 days in the dark and at room temperature. Sizing was performed by DLS and TEM, as above. For TEM, nanoparticles were thoroughly washed with double distilled water to remove excess PVA beforehand. The Gemcitabine loading in the PLGem was determined by UV-visible spectroscopy at the wavelength λ = 268 nm. This also allowed us to calculate the encapsulation efficiency (EE) for PLGem as follows:

(2) $$\text{EE}=\left(\text{mass encapsulated Gemcitabine}\right)/\left(\text{mass starting material Gemcitabine}\right)\;\text{X}\;100$$

### Transmission electron microscopy (TEM) of the nanoparticles

Samples were deposited onto a carbon membrane supported by a copper grid and left until complete drying was achieved. Next, a drop of 2% uranyl acetate solution was applied to improve the contrast of the sample. TEM observations were performed on a JEOL JEM 200 CX microscope for PLGem, achieving a lattice and a point-to-point resolution of 1.4 Å and 3.5 Å. The acceleration voltage was set to 120 kV. GemPo was imaged with a JEOL 1200-EX operating at 80 kV.

### In vitro drug release profiles

PLGem or GemPo were suspended in 500 μL of PBS or PANC1 cell lysates, and sealed in a dialysis bag (MWCO ≈ 1,000 Da). The dialysis bag was incubated in 1 mL of PBS buffer at room temperature with gentle shaking, in a humidified chamber to prevent evaporation, and the dialysis was kept for up to a month. 10 μL of aliquots were extracted from the incubation medium at predetermined time intervals, dissolved in 90 μL DMSO and the released Gemcitabine was quantified by UV-visible spectroscopy at the characteristic wavelength of λ = 268 nm. After withdrawing each aliquot, the incubation medium was replenished with 10 μL of fresh PBS.

### Cell culture

The human pancreatic carcinoma cell line, PANC1, was obtained from American Type Tissue Culture Collection (Rockville, MD) and was maintained in DMEM supplemented with 10% FBS and antibiotic/antimycotic (all from Invitrogen, Carlsbad, CA). PANC1 cells were grown on 100 mm dishes, subcultured using trypsin (0.25%) and EDTA (0.01%) treatment and replated at 2,500 cells.cm^-2^. Cells were incubated with serum-deprived medium prior to drug addition, which itself was in complete medium. For all experiments, cells were treated with PLGem, GemPo or free Gemcitabine, with solvent- or empty nanovector-treated cells serving as internal controls.

### MTS cytotoxicity assay

PANC1 cells were seeded at a density of 10,000 cells per well in 96-well plates overnight. Cells were incubated with drugs for 3 days. Solvent-treated cells served as internal controls. The percentages of viable cells were then quantified with 3-(4,5-Dimethylthiazol-2-yl)-5-(3-carboxymethoxyphenyl)-2-(4-sulfophenyl)-2 H-tetrazolium (MTS) from the CellTiter 96 AQueous One Solution kit (Promega Corporation, Madison, WI). MTS is reduced by mitochondrial dehydrogenases of live cells, yielding a colored adduct that can be read spectrophotometrically. Briefly, the cells were washed with PBS, incubated with 0.3 mg.mL^-1^ of MTS, in basal medium without phenol red, for 2 h at 37°C and absorbance was then measured at 490 nm in a microplate spectrophotometer (Epoch, Biotek Instruments, Winooski, VT). Final absorbance, corresponding to cell proliferation, was plotted after removing background values from each data point and divided by the mean of solvent-treated cells.

### Apoptosis study by AnnexinV-FITC and propidium iodide staining

Cells grown in 6-well plates were treated with 1 μM drugs for 2 days, and incubated with 5 μL of AnnexinV-Alexa Fluor 488 in binding buffer (10 mM HEPES, 140 mM NaCl, 2.5 mM CaCl_2_, pH 7.4) for 15 min in the dark, according to the manufacturer’s protocol. Cells were then washed with binding buffer, counterstained with propidium iodide and immediately processed for FITC and propidium iodide detection using an Accuri C6 flow cytometer (ex/em 488/499 and 535/617 nm, respectively). AnnexinV-Alexa Fluor 488, propidium iodide, or both, were omitted for the negative controls.

### Ultracharacterization studies of pancreatic cells by TEM

PANC1 cells treated with 1 μM of drugs for 1 day were fixed in 2.5% gluteraldehyde, 1.25% paraformaldehyde and 0.03% picric acid in 0.1 M sodium cacodylate buffer (pH = 7.4). The cells were then postfixed for 30 min in 1% Osmium tetroxide (OsO_4_)/1.5% Potassium ferrocyanide (K_4_Fe(CN)_6_), washed in water 3 times and incubated in 1% aqueous uranyl acetate for 30 min, followed by 2 washes in water and subsequent dehydration in grades of alcohol (5 min each: 50%, 70%, 95%, 2x 100%). Cells were removed from the dish in propyleneoxide, pelleted at 3,000 rpm for 3 min and infiltrated for 2 h in a 1:1 mixture of propyleneoxide and TAAB Epon (Marivac Canada Inc. St. Laurent, Canada). The samples were subsequently embedded in TAAB Epon and polymerized at 60°C for 2 days. Ultrathin sections (about 60 nm) were cut on a Reichert Ultracut-S microtome, picked up on to copper grids stained with lead citrate and examined in a Tecnai G^2^ Spirit BioTWIN microscope operating at 80 kV and images were recorded with an AMT 2 k CCD camera. Solvent- and empty nanovector-treated cells served as internal controls.

### Statistical analysis

All results were expressed as mean ± SEM of at least quadruplate samples. Statistical comparisons were obtained using one-way ANOVA, followed by the Newman-Keuls test. Probability (p) values less than 0.05 were considered significant.

## Results

### Physico-chemical parameters of PLGem and GemPo

#### Synthesis of Gemcitabine-loaded nanoplatforms

**Figure 1:**
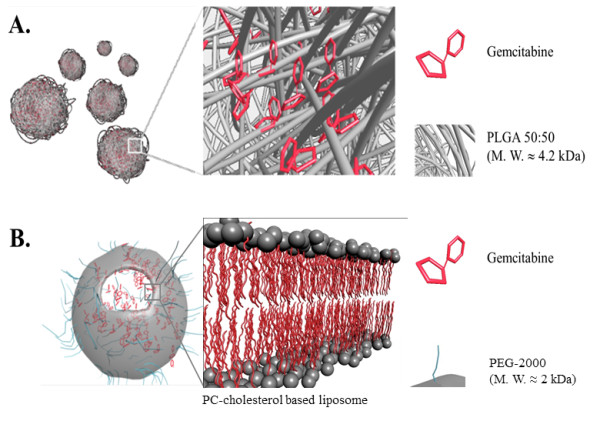
**Schematic representations of Gemcitabine encapsulation into two biodegradable nanovectors.** (**A**) PLGem is defined as Gemcitabine encapsulated in poly(lactic-co-glycolic) acid (PLGA) nanoparticles having a molecular weight of around 4.2 kDa by emulsion-evaporation technique. Magnification shows the predicted arrangement of Gemcitabine. (**B**) GemPo refers to Gemcitabine encapsulated in phosphatidylcholine-based liposomes using the lipid film hydration technique. Magnification shows the lipid bilayer.

Gemcitabine was entrapped into two different types of biodegradable nanovectors widely used in the nanotechnology field, namely PLGA polymer (Figure 1A) or cholesterol-based liposome (Figure 1B). We termed these drugs *PLGem* and *GemPo*, respectively. PLGem was generated using the emulsion-solvent evaporation technique, whereas GemPo was synthesized using lipid film hydration. Due to the mainly hydrophilic property of Gemcitabine, it is hypothesized to be preferentially integrated in the aqueous core of the liposome, whereas Gemcitabine should predominantly interact with PLGA through non-covalent interactions, as depicted in Figure 1.

#### Nanoparticles morphologies, sizes and encapsulation efficiencies

**Figure 2:**
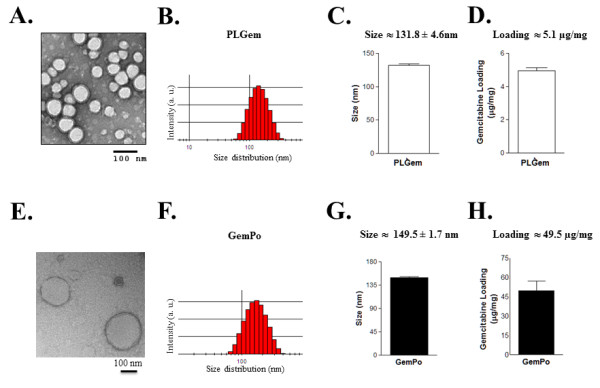
**Physico-chemical characterizations of PLGem and GemPo.** Transmission Electron Microscopy (TEM) images of (**A**) PLGem and (**E**) GemPo and their corresponding size distribution (**B** and **F**, respectively) and mean size histograms (**C** and **G**, respectively) as detected by Dynamic Light Scattering (DLS). Gemcitabine loading onto (**D**) PLGA and into (H) liposomes are graphed based on UV-visible spectroscopy at λ = 268 nm.

The morphology and size distribution of the nanoparticles were evaluated by TEM (Figure 2A and E) and DLS (Figure 2B and F), respectively. TEM demonstrated that spherical nanoparticles were obtained for both PLGem and GemPo. DLS showed a mean size distribution of 131.8 ± 4.6 and 149.5 ± 1.7 nm in diameter for PLGem (Figure 2C) and GemPo (Figure 2G), respectively, sizes at which increased cell internalization is known to occur [26]. Although PLGem and GemPo exhibited similar sizes, their surface charges significantly differed, as assessed by their zeta potential values (− 31.1 ± 9.8 mV for PLGem and - 4.3 ± 6.6 mV for GemPo), thus showing the inherent distinctive properties of each vectors (results are not shown). The loading of Gemcitabine in each vector was determined by UV-visible spectroscopy at its characteristic emission wavelength of λ = 268 nm, yielding 5.1 μg/mg for PLGem (Figure 2D) and 49.5 μg/mg for GemPo (Figure 2H), which translates to encapsulation efficiencies of 10% (PLGem) and 35% (GemPo). Gemcitabine, as other water soluble drugs, lead to low encapsulation efficiency in PLGA and liposome nanoparticles as compared with lipophilic drugs [29,30]. Encouragingly, PLGem loading was still 1.7-fold higher than what we previously published with regards to the encapsulation of another drug in PLGA nanoparticles [24].

#### Gemcitabine release profiles are altered by nanovector type

**Figure 3:**
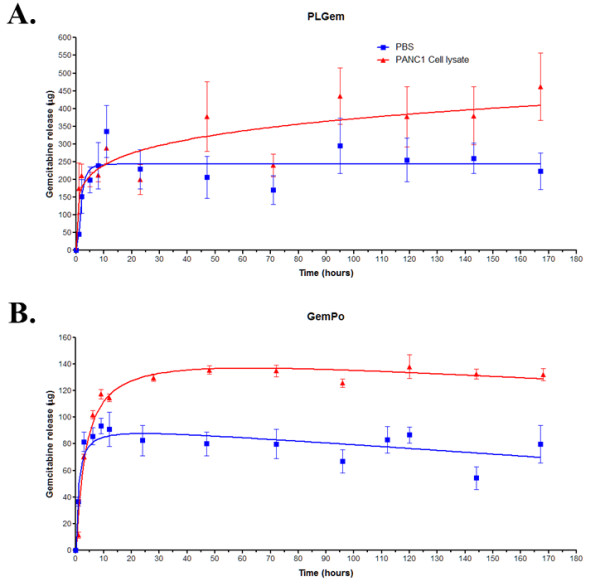
**Gemcitabine release profiles from PLGem and GemPo.** Gemcitabine release profiles from (**A**) PLGem and (**B**) GemPo nanoparticles were evaluated in PBS or human pancreatic carcinoma (PANC1) cell lysates. Values on the Y-axis represent the amount of Gemcitabine released from the nanoparticles in a defined volume.

The *in vitro* Gemcitabine release profiles from PLGem (Figure 3A) and GemPo (Figure 3B) were assessed in an aggressive human pancreatic carcinoma cell line, PANC1, which is well-known to exhibit Gemcitabine resistance, thus serving as an ideal model to investigate the roles of PLGem and GemPo in PDA [9]. As shown in Figure 3, PLGem and GemPo exhibited burst release of Gemcitabine in both PANC1 lysates and PBS, which peaked faster with regards to GemPo (28 h, Figure 3B) than with PLGem (47 h, Figure 3A), thus confirming the characteristic burst release profile associated with nanoparticle encapsulation [31]. Both PLGem and GemPo achieved sustained release of Gemcitabine for at least 7 days, consistent with the temporal control imparted by nanoparticles on drugs [16]. Furthermore, there was a marked difference in the release profiles between both nanoplatforms, with PLGem delivering about 3-fold more payload than GemPo, which translated to around 95% and 33% release, respectively, over the tested period of time. Differences were also observed with respect to both nanoplatforms and their microenvironment: whereas an almost 2-fold Gemcitabine release was observed in PANC1 versus PBS in GemPo, the release profile from PLGem was only slightly higher in the cancer cell lysate than in PBS.

### Biological studies

#### Gemcitabine-mediated cytotoxicity is improved in the PLGA nanovector

**Figure 4:**
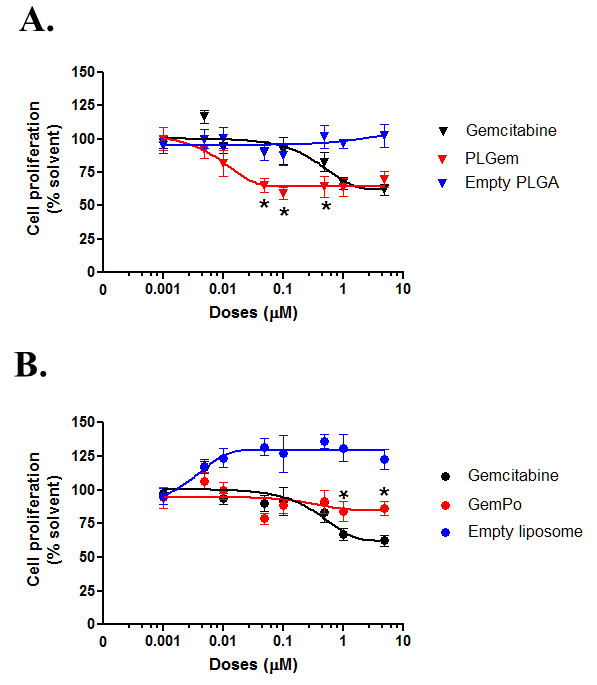
**Cytotoxic profiles of PLGem and GemPo on PANC1 cells.** (**A**) PLGem’s and (**B**) GemPo’s effects on human pancreatic carcinoma cell proliferation were determined by MTS assay. PANC1 cells were plated on 96-well plates in the presence or absence of either (**A**) empty PLGA vehicle, free drug (Gemcitabine) and PLGem or (**B**) empty liposome vehicle, free Gemcitabine and GemPo, in a concentration-dependent manner. After three days, the proportion of live cells remaining were quantified and plotted as percentage of solvent-treated cells. Legend is indicated in inset. *p <; 0.05 between nanoparticle and free drug.

To assess whether Gemcitabine release is prolonged in both nanoformulations, we next measured the cytotoxicity profiles of PLGem and GemPo versus that of free Gemcitabine in PANC1 cells using the MTS assay (Figure 4). Data was normalized to solvent-treated cells and empty PLGA or empty liposome were tested to exclude any nanovector-based artefacts. These latter controls are crucial to understanding the encapsulation efficiency of a drug, yet have been omitted from most reports thus far. After 3 days, there was a significant difference between PLGem versus free Gemcitabine from 0.05 μM to 0.5 μM (Figure 4A). For instance, 91.5 (Gemcitabine) versus 59.1% (PLGem) of cells remained viable at 0.1 μM. On the contrary in the case of GemPo, free Gemcitabine was slightly more potent, with 62.2 (Gemcitabine) and 86.3% (GemPo) viable cells remaining after treatment with 5 μM drugs (Figure 4B). These results indicate that Gemcitabine release is more prolonged in PLGA nanovector, as compared with its liposomal counterpart.

#### Gemcitabine-mediated apoptosis is improved in the PLGA nanovector

**Figure 5:**
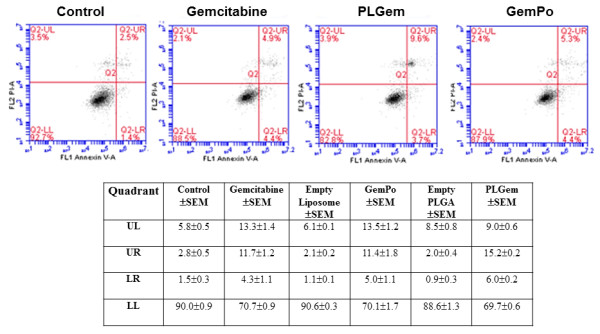
**Apoptotic profiles of PLGem and GemPo on PANC1 Cells.** The percentage of early and late apoptotic cells was quantified by the AnnexinV-propidium iodide flow cytometry method. PANC1 cells treated with free Gemcitabine (Gem) or Gemcitabine nanoformulations (PLGem or GemPo) for 2 days were subjected to FACS analysis. Cells were gated into four quadrants so as to measure viable cells: LL (lower left), early apoptosis: LR (lower right) and late apoptosis: UR (upper right). The percentage of cells per quadrant is depicted on the bottom and representative examples are shown on top.

The best-characterized underlying mechanism of Gemcitabine is its pro-apoptotic effect. To determine whether there is a difference in the induction of apoptosis between PLGem, GemPo and free drug, the apoptotic profiles of PANC1 cells were investigated using the AnnexinV-propidium iodide fluorescence activated cell sorting (FACS) assay. Figure 5 shows that GemPo treatment resulted in slightly higher early apoptosis than Gemcitabine (5.0 and 4.3% of cell death, respectively), whereas both treatments elicited similar effects during late apoptosis (11.4 and 11.7% cell death, respectively). In contrast, PLGem treatment shifted the apoptotic response to 6.0 (early) and 15.2% (late), corroborating the MTS data from Figure 4.

#### Ultracharacterization studies show differing internalization mechanisms between free Gemcitabine, PLGem and GemPo

**Figure 6:**
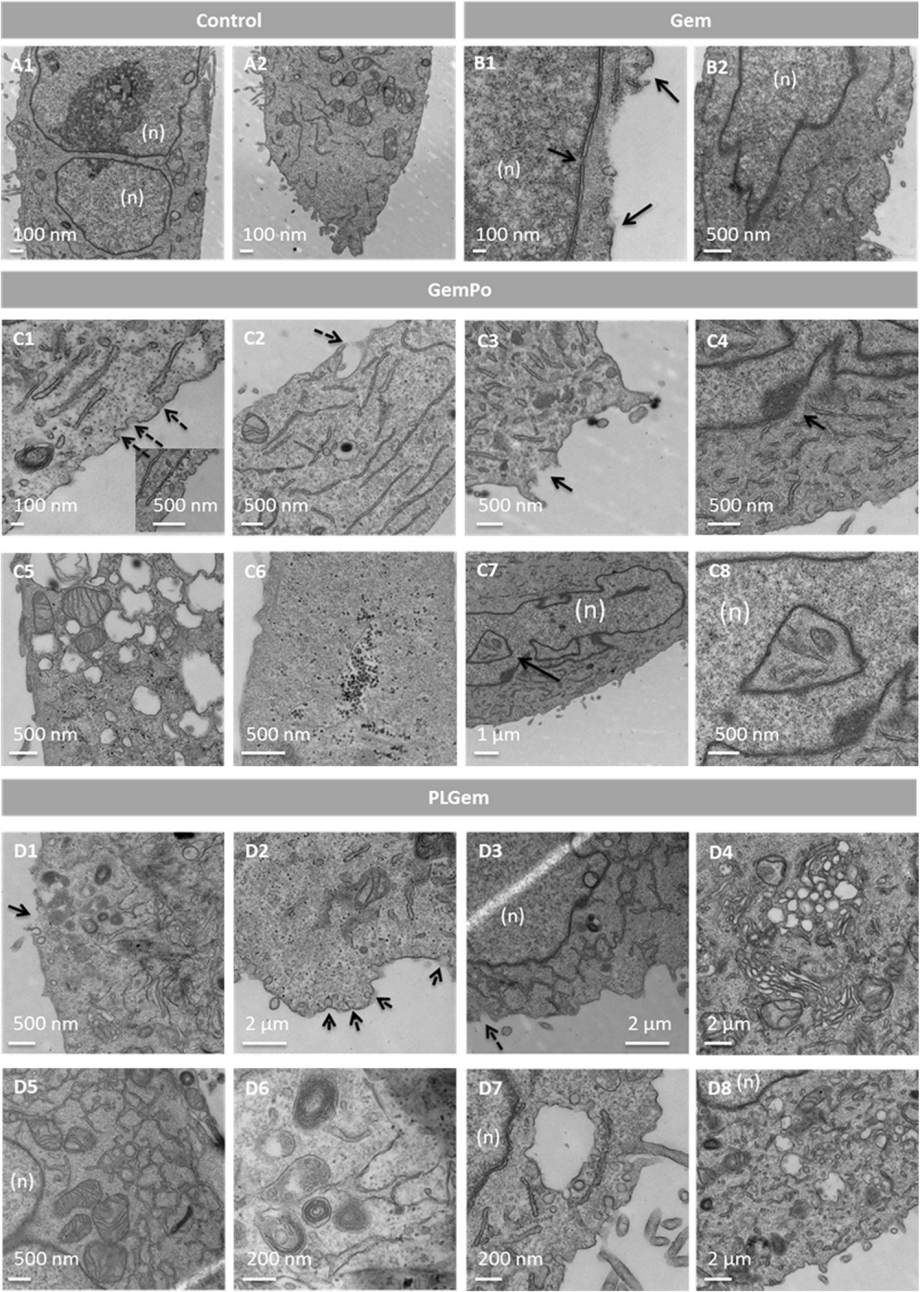
**Ultracharacterization studies of Gemcitabine, PLGem and GemPo in PANC1 cells.** Cells were left (**A**) untreated, or treated with (**B**) free Gemcitabine, (**C**) GemPo or (**D**) PLGem for 1 day and then processed for TEM analysis. (n) indicates nucleus localization. Full and doted arrows highlight cytosolic and uptake pathways, respectively.

To elucidate the underlying mechanisms mediating Gemcitabine’s intracellular trafficking, ultracharacterization studies using TEM were carried out in PANC1 cells incubated with the free drug or Gemcitabine nanoformulations for 1 day and compared with the control (Figures 6A1, A2). As shown in Figure 6, all Gemcitabine formulations caused severe perturbations of the plasma and nuclear membranes, although to different extents. Effectively, endocytosis with the free drug employed small invaginations not exceeding 100 nm in size (Figures 6B1, B2), whereas both nanoplatforms employed larger invaginations around 500 nm in diameter (Figures 6C2, C3 for GemPo, 6D1, D3 for PLGem). This suggests that nanovectors underwent clathrin-mediated endocytosis whereas the free drug was taken up by caveolae [32]. As for the nucleus, membranal fenestrations of up to 100 nm were observed for all groups, which correspond to an almost 3-fold increase in size as compared to the standard nuclear pore size (Figures 6B1, C4, D7) [33], indicating Gemcitabine had reached its nuclear target. These fenestrations were slightly more pronounced for Gemcitabine nanoplatforms.

There were significant differences between free Gemcitabine and both nanoplatforms with respect to off-target effects. Only PLGem- and GemPo-treated cells showed significant dilatation/swelling of the endoplasmic reticulum (ER) (Figures 6C5 and D4), which correlated with an overproduction of glycogen, localizing to the cytoplasm for PLGem (Figure 6D2) and to the cytoplasm, ribosomes and vesicles for GemPo (Figures 6C1 and C6). In addition, significant loss of electron density in the cytoplasm was observed for both nanoplatforms (Figures 6C2, D6). Lastly, only GemPo (Figures 6C7, C8) and PLGem (results not shown) treatments significantly enhanced mitochondrial pseudo-inclusions into the nucleus.

Differences between both nanoplatforms were also observed, as PLGem caused additional perturbations. As such, PLGem treatment led to an intracellular trafficking pathway not observed with GemPo, namely the presence of larger transport vesicles (mean size around 2μm, figure 6D7) adjacent to the membranal invaginations and leading up to the nucleus (Figure 6D8). PLGem also provoked a strong morphological perturbation of mitochondrial cristae into a concentric configuration (Figure 6D6), as well as ER hypertrophy (Figure 6D5).

## Discussion

Nanotechnology has made immense progress in the last decade, providing novel treatments as a last resort for hard-to-treat cancers, and more recently, in the pancreatic cancer field [15]. An advantage of nanotechnology lies in the ability to engineer tailor-made formulations to meet ones need. However, the underlying mechanisms explaining the beneficial effect of nanoplatforms versus those of free drugs on drug trafficking remain unclear, as are the effects of the nanovectors themselves. Elucidating these mechanisms could provide crucial insights into engineering novel cancer therapeutics yielding more potent and selective nanoformulations. In the current study, we investigated the mechanisms of free Gemcitabine and two Gemcitabine nanoplatforms, namely *PLGem* and *GemPo*, in a resistant human pancreatic cancer cell line, PANC1, which most closely mimics the PDA phenotype and hence, is ideally suited to investigate the role of nanovectors in circumventing drug resistance [9]. In non-resistant pancreatic cells, our preliminary data shows that Gemcitabine and the nanoparticles elicited almost complete cell death, hence making the comparison between free and encapsulated Gemcitabine difficult. Our data also indicates that this inherent resistance pertains to the presence of membrane efflux pumps selectively in the PANC1 cell line. The exact mechanisms by which nanoparticles are able to evade this resistance, however, remains to be investigated.

In order for nanoparticles to deliver proper payload and thus achieve pharmacological effects, drug release and subsequent sustained degradation of each nanoplatform are critical steps [15]. The rates of degradation mainly depend on diffusion of drugs through the nanovectors, as well as erosion of these nanoparticles [34]. Based on the Gemcitabine release profiles studies herein, although both nanoplatforms exhibited sustained drug release, PLGem delivered a higher payload than GemPo in PANC1 cells, which correlated with the increased cytotoxicity and apoptosis observed with PLGem. Higher hydrolytic degradation rates reported for PLGA backbones as opposed to lipids have previously been reported [35,36]. These results indicate that PLGA is a more-suited nanovector for administering Gemcitabine to resistant pancreatic cancer cells as it preferentially prolongs Gemcitabine’s release.

Thus far, studies which investigated the mechanisms of nanoparticle internalization in cancer cells mainly focused on endocytotic mechanisms including clathrin-mediated endocytosis, which is supported in this study [37], and phagocytosis, [19,38,39]. However, subsequent intracellular trafficking studies were only based on inferences from biological assays (e.g. MTS, apoptosis), or labeling of the nanovector with a fluorochrome. Although the later approach successfully allows monitoring of nanovector internalization in real-time, it does not take into account that the drug has already been release from the nanovector, nor does it allow for precise investigation of the drug’s effect on organelles. In this study, we have overcome these limitations by performing ultrastructural analysis in order to track Gemcitabine (free or encapsulated) internalization inside PANC1 cells.

Ultrastructural analysis provides a straightforward mean of investigating whole-cell effects of Gemcitabine at high resolution. Effectively, both on-target (eg. nuclear) and off-target (eg. ER, cytoplasmic and mitochondrial) differences in intracellular trafficking between treatment groups were readily observed. As such, TEM investigation shows that free drug was internalized by a 5-fold smaller membranal invaginations than both nanoplatforms, indicating that an additional internalization pathway occurs in the presence of the nanovectors, which is supported by several studies [38,40]. Furthermore, the degree of nuclear fenestrations implies that PLGem and GemPo reach their nuclear target more robustly than Gemcitabine, which might explains why GemPo treatment yielded slightly more apoptosis.

Mechanisms proper to the nanoplatforms include significant mitochondrial pseudo-inclusions into the nucleus, dilatation/swelling of the ER and loss of electron density in the cytoplasm. These effects have been reported in instances of severe apoptosis and necrosis [41-44]. Interestingly, there were major differences between both nanoplatforms, as PLGem elicited more off-target effects than GemPo. PLGem administration resulted in the appearance of large vacuoles adjacent to the membranal invaginations and leading up to the nucleus, implying a more robust pathway by which PLGem reaches its genomic target as opposed to GemPo. PLGem treatment also led to significant ER hypertrophy and rearrangement of mitochondrial cristae into concentric rings. These cristae rearrangements are thought to be a defense mechanism during instances of cellular deterioration, and have been reported in a case of hypertrophic cardiomyopathy [45,46]. Furthermore, the appearance of ER hypertrophy reflects a more advanced state of ER stress and apoptosis as opposed to GemPo treatment [47], which is corroborated by both MTS and FACS data. These results constitute, to the best of our knowledge, novel findings, and might represent a strategy deployed by PLGem to overcome drug resistance in resistant pancreatic cells, although this remains to be investigated.

Taken together, ultrastructural mechanistic studies in the PANC1 cell line confirm the preferential biological effects observed with PLGem *versus* GemPo and free Gemcitabine and further indicate that, when Gemcitabine is delivered in PLGA, it can more potently target several organelles asides from the nucleus, including the ER and mitochondria.

## Conclusions

In conclusion, our study uncovers novel mechanisms of action employed by Gemcitabine-loaded nanoplatforms, as opposed to free drug, and confirms that the choice of nanovector is a crucial parameter that should be taken into consideration for delivering Gemcitabine to resistant pancreatic cancer cells. Although these results need to be validated *in vivo*, they represent the first study of its kinds in pancreatic cancer research and could serve as an intermediate step before passing to mice studies. Interestingly, the ultrastructural analyses reported herein uncovered potential new targets which could be combined with Gemcitabine for pancreatic cancer treatment, namely the ER and mitochondria.

## Abbreviations

DLS: Dynamic Light Scattering; EE: Encapsulation Efficiency; ER: Endoplasmic Reticulum; FACS: Fluorescence Activated Cell Sorting; MTS: 3-(4,5-Dimethylthiazol-2-yl)-5-(3-carboxymethoxyphenyl)-2-(4-sulfophenyl)-2 H-tetrazolium; PDA: Pancreatic Ductal Adenocarcinoma; PLGA: Poly(lactic-co-glycolic acid); TEM: Transmission Electron Microscopy.

## Competing interests

The authors declare that they have no competing interests.

## Authors’ contributions

Conceived and designed the experiments: RH ALP SB PS. Performed the experiments: RH ALP DB. Analyzed the data: RH ALP. Contributed reagent/material/analysis tools: SS and RH. Wrote the paper: RH ALP. All authors read and approved the final manuscript.

## Pre-publication history

The pre-publication history for this paper can be accessed here:

http://www.biomedcentral.com/1471-2407/12/419/prepub

## References

[B1] Merl MY Li J Saif MW The first-line treatment for advanced pancreatic cancer. Highlights from the "2010 ASCO Gastrointestinal Cancers Symposium JOP 2010 11 148 150 Orlando, FL, USA. January 22–24 20208324

[B2] Pliarchopoulou K Pectasides D Pancreatic cancer: Current and future treatment strategies Cancer Treat Rev 2009 35 431 436 10.1016/j.ctrv.2009.02.005 19328630

[B3] Harsha HC Kandasamy K Ranganathan P Rani S Ramabadran S Gollapudi S Balakrishnan L Dwivedi SB Telikicherla D Selvan LD Goel R Mathivanan S Marimuthu A Kashyap M Vizza RF Mayer RJ Decaprio JA Srivastava S Hanash SM Hruban RH Pandey A A compendium of potential biomarkers of pancreatic cancer PLoS Med 2009 6 e1000046 10.1371/journal.pmed.1000046 19360088 PMC2661257

[B4] Yachida S Iacobuzio-Donahue CA The pathology and genetics of metastatic pancreatic cancer Arch Pathol Lab Med 2009 133 413 422 19260747 10.5858/133.3.413

[B5] Di MM Di CR Macchini M Nobili E Vecchiarelli S Brandi G Biasco G Metastatic pancreatic cancer: is gemcitabine still the best standard treatment? (Review) Oncol Rep 2010 23 1183 1192 20372829 10.3892/or_00000749

[B6] Patra CR Bhattacharya R Wang E Katarya A Lau JS Dutta S Muders M Wang S Buhrow SA Safgren SL Yaszemski MJ Reid JM Ames MM Mukherjee P Mukhopadhyay D Targeted delivery of gemcitabine to pancreatic adenocarcinoma using cetuximab as a targeting agent Cancer Res 2008 68 1970 1978 10.1158/0008-5472.CAN-07-6102 18339879

[B7] Hidalgo M Pancreatic cancer N Engl J Med 2010 362 1605 1617 10.1056/NEJMra0901557 20427809

[B8] Bornmann C Graeser R Esser N Ziroli V Jantscheff P Keck T Unger C Hopt UT Adam U Schaechtele C von DE Massing U A new liposomal formulation of Gemcitabine is active in an orthotopic mouse model of pancreatic cancer accessible to bioluminescence imaging Cancer Chemother Pharmacol 2008 61 395 405 10.1007/s00280-007-0482-z 17554540

[B9] Huanwen W Zhiyong L Xiaohua S Xinyu R Kai W Tonghua L Intrinsic chemoresistance to gemcitabine is associated with constitutive and laminin-induced phosphorylation of FAK in pancreatic cancer cell lines Mol Cancer 2009 8 125 10.1186/1476-4598-8-125 20021699 PMC2806309

[B10] Kleynberg RL Sofi AA Chaudhary RT Hand-Foot Hyperpigmentation Skin Lesions Associated With Combination Gemcitabine-Carboplatin (GemCarbo) Therapy Am J Ther 2010 18 e261 e263 20460984 10.1097/MJT.0b013e3181d860f6

[B11] Hong SP Wen J Bang S Park S Song SY CD44-positive cells are responsible for gemcitabine resistance in pancreatic cancer cells Int J Cancer 2009 125 2323 2331 10.1002/ijc.24573 19598259

[B12] Reid JM Qu W Safgren SL Ames MM Krailo MD Seibel NL Kuttesch J Holcenberg J Phase I trial and pharmacokinetics of gemcitabine in children with advanced solid tumors J Clin Oncol 2004 22 2445 2451 10.1200/JCO.2004.10.142 15197207

[B13] Rapoport N Kennedy AM Shea JE Scaife CL Nam KH Ultrasonic nanotherapy of pancreatic cancer: lessons from ultrasound imaging Mol Pharm 2010 7 22 31 10.1021/mp900128x 19899813 PMC2815246

[B14] Pedersen AG Phase I studies of gemcitabine combined with carboplatin or paclitaxel Semin Oncol 1997 24 S7 9194483

[B15] Moghimi SM Hunter AC Murray JC Nanomedicine: current status and future prospects FASEB J 2005 19 311 330 10.1096/fj.04-2747rev 15746175

[B16] Davis ME Chen ZG Shin DM Nanoparticle therapeutics: an emerging treatment modality for cancer Nat Rev Drug Discov 2008 7 771 782 10.1038/nrd2614 18758474

[B17] Stavridi F Palmieri C Efficacy and toxicity of nonpegylated liposomal doxorubicin in breast cancer Expert Rev Anticancer Ther 2008 8 1859 1869 10.1586/14737140.8.12.1859 19046106

[B18] Couvreur P Vauthier C Nanotechnology: intelligent design to treat complex disease Pharm Res 2006 23 1417 1450 10.1007/s11095-006-0284-8 16779701

[B19] Kim JS Yoon TJ Yu KN Noh MS Woo M Kim BG Lee KH Sohn BH Park SB Lee JK Cho MH Cellular uptake of magnetic nanoparticle is mediated through energy-dependent endocytosis in A549 cells J Vet Sci 2006 7 321 326 10.4142/jvs.2006.7.4.321 17106221 PMC3242138

[B20] Pili B Reddy LH Bourgaux C Lepetre-Mouelhi S Desmaele D Couvreur P Liposomal squalenoyl-gemcitabine: formulation, characterization and anticancer activity evaluation Nanoscale 2010 2 1521 1526 10.1039/c0nr00132e 20820745

[B21] Papa AL Dumont L Vandroux D Millot N Titanate nanotubes: towards a novel and safer nanovector for cardiomyocytes Nanotoxicology 2012 in press 10.3109/17435390.2012.710661 22770363

[B22] Sengupta S Eavarone D Capila I Zhao G Watson N Kiziltepe T Sasisekharan R Temporal targeting of tumour cells and neovasculature with a nanoscale delivery system Nature 2005 436 568 572 10.1038/nature03794 16049491

[B23] Basu S Harfouche R Soni S Chimote G Mashelkar RA Sengupta S Nanoparticle-mediated targeting of MAPK signaling predisposes tumor to chemotherapy Proc Natl Acad Sci USA 2009 106 7957 7961 10.1073/pnas.0902857106 19383798 PMC2683142

[B24] Harfouche R Basu S Soni S Hentschel DM Mashelkar RA Sengupta S Nanoparticle-mediated targeting of phosphatidylinositol-3-kinase signaling inhibits angiogenesis Angiogenesis 2009 12 325 338 10.1007/s10456-009-9154-4 19685150

[B25] Celano M Calvagno MG Bulotta S Paolino D Arturi F Rotiroti D Filetti S Fresta M Russo D Cytotoxic effects of gemcitabine-loaded liposomes in human anaplastic thyroid carcinoma cells BMC Cancer 2004 4 63 10.1186/1471-2407-4-63 15363094 PMC517941

[B26] Cosco D Bulotta A Ventura M Celia C Calimeri T Perri G Paolino D Costa N Neri P Tagliaferri P Tassone P Fresta M In vivo activity of gemcitabine-loaded PEGylated small unilamellar liposomes against pancreatic cancer Cancer Chemother Pharmacol 2009 64 1009 1020 10.1007/s00280-009-0957-1 19263052

[B27] Cosco D Paolino D Cilurzo F Casale F Fresta M Gemcitabine and tamoxifen-loaded liposomes as multidrug carriers for the treatment of breast cancer diseases Int J Pharm 2012 422 229 237 10.1016/j.ijpharm.2011.10.056 22093954

[B28] Trickler WJ Khurana J Nagvekar AA Dash AK Chitosan and glyceryl monooleate nanostructures containing gemcitabine: potential delivery system for pancreatic cancer treatment AAPS PharmSciTech 2010 11 392 401 10.1208/s12249-010-9393-0 20238190 PMC2850475

[B29] Tewes F Munnier E Antoon B Ngaboni OL Cohen-Jonathan S Marchais H Douziech-Eyrolles L Souce M Dubois P Chourpa I Comparative study of doxorubicin-loaded poly(lactide-co-glycolide) nanoparticles prepared by single and double emulsion methods Eur J Pharm Biopharm 2007 66 488 492 10.1016/j.ejpb.2007.02.016 17433641

[B30] Niu G Cogburn B Hughes J Preparation and characterization of doxorubicin liposomes Methods Mol Biol 2010 624 211 219 10.1007/978-1-60761-609-2_14 20217598

[B31] Sunoqrot S Bae JW Jin SE Pearson M Liu Y Hong S Kinetically controlled cellular interactions of polymer-polymer and polymer-liposome nanohybrid systems Bioconjug Chem 2011 22 466 474 10.1021/bc100484t 21344902 PMC3059376

[B32] Canton I Battaglia G Endocytosis at the nanoscale Chem Soc Rev 2012 41 2718 2739 10.1039/c2cs15309b 22389111

[B33] Alber F Dokudovskaya S Veenhoff LM Zhang W Kipper J Devos D Suprapto A Karni-Schmidt O Williams R Chait BT Sali A Rout MP The molecular architecture of the nuclear pore complex Nature 2007 450 695 701 10.1038/nature06405 18046406

[B34] Soppimath KS Aminabhavi TM Kulkarni AR Rudzinski WE Biodegradable polymeric nanoparticles as drug delivery devices J Control Release 2001 70 1 20 10.1016/S0168-3659(00)00339-4 11166403

[B35] Hasirci V Lewandrowski K Gresser JD Wise DL Trantolo DJ Versatility of biodegradable biopolymers: degradability and an in vivo application J Biotechnol 2001 86 135 150 10.1016/S0168-1656(00)00409-0 11245902

[B36] Reddy KR Controlled-release, pegylation, liposomal formulations: new mechanisms in the delivery of injectable drugs Ann Pharmacother 2000 34 915 923 10928404 10.1345/aph.10054

[B37] Paillard A Hindre F Vignes-Columbeix C Benoit JP Garcion E The importance of endo-lysosomal escape with lipid nanocapsules for drug subcellular bioavailability Biomaterials 2010 31 7542 7554 10.1016/j.biomaterials.2010.06.024 20630585

[B38] Resina S Prevot P Thierry AR Physico-chemical characteristics of lipoplexes influence cell uptake mechanisms and transfection efficacy PLoS One 2009 4 e6058 10.1371/journal.pone.0006058 19557145 PMC2699663

[B39] Dandekar P Jain R Stauner T Loretz B Koch M Wenz G Lehr CM A Hydrophobic Starch Polymer for Nanoparticle-Mediated Delivery of Docetaxel Macromol Biosci 2011 12 184 194 22127828 10.1002/mabi.201100244

[B40] Rejman J Oberle V Zuhorn IS Hoekstra D Size-dependent internalization of particles via the pathways of clathrin- and caveolae-mediated endocytosis Biochem J 2004 377 159 169 10.1042/BJ20031253 14505488 PMC1223843

[B41] Bakeeva LE Skulachev VP Sudarikova YV Tsyplenkova VG Mitochondria enter the nucleus (one further problem in chronic alcoholism) Biochemistry (Mosc) 2001 66 1335 1341 10.1023/A:1013374410540 11812238

[B42] Vaquero EC Rickmann M Molero X Tocotrienols: balancing the mitochondrial crosstalk between apoptosis and autophagy Autophagy 2007 3 652 654 17932465 10.4161/auto.5088

[B43] Donadelli M Dando I Zaniboni T Costanzo C Dalla PE Scupoli MT Scarpa A Zappavigna S Marra M Abbruzzese A Bifulco M Caraglia M Palmieri M Gemcitabine/cannabinoid combination triggers autophagy in pancreatic cancer cells through a ROS-mediated mechanism Cell Death Dis 2011 2 e152 10.1038/cddis.2011.36 21525939 PMC3122066

[B44] Fujita R Ueda H Protein kinase C-mediated necrosis-apoptosis switch of cortical neurons by conditioned medium factors secreted under the serum-free stress Cell Death Differ 2003 10 782 790 10.1038/sj.cdd.4401239 12815461

[B45] Ghadially FN Ultrastructural Pathology of the Cell and Matrix 1997 4 Butterworth-Heineman, Boston 1

[B46] Kanzaki Y Terasaki F Okabe M Otsuka K Katashima T Fujita S Ito T Kitaura Y Giant mitochondria in the myocardium of a patient with mitochondrial cardiomyopathy: transmission and 3-dimensional scanning electron microscopy Circulation 2010 121 831 832 10.1161/CIR.0b013e3181d22e2d 20159843

[B47] Meares GP Mines MA Beurel E Eom TY Song L Zmijewska AA Jope RS Glycogen synthase kinase-3 regulates endoplasmic reticulum (ER) stress-induced CHOP expression in neuronal cells Exp Cell Res 2011 317 1621 1628 10.1016/j.yexcr.2011.02.012 21356208 PMC3103628

